# Infected Schmorl’s node: a case report

**DOI:** 10.1186/s12891-020-03276-4

**Published:** 2020-05-02

**Authors:** Hyeun Sung Kim, Harshavardhan Dilip Raorane, Sagar Bhupendra Sharma, Pang Hung Wu, Il-Tae Jang

**Affiliations:** Department of Neurosurgery, Nanoori Hospital Gangnam, 731, Eonju-ro, Gangnam-gu, Seoul, 06048 Republic of Korea

**Keywords:** Schmorl’s node, Symptomatic, Infected, MRI

## Abstract

**Background:**

Schmorls node (SN) are mostly asymptomatic and incidental findings on MRI. However, sometimes they present like acute onset low back pain or acute exacerbation of chronic back pain after minor trauma.

**Case presentation:**

We present rare case of symptomatic infected SN in 67 years female patient presented with complains of low back pain radiating to right buttock. After initial conservative treatment failed subsequent imaging showed significant increase in size of lesion with focal signal changes in disc space gave suspicion of underlying secondary pathology. Patient operated for complete excision of lesion. Histopathological report was suggestive of pyogenic vertebral osteomyelitis. Patient improved well postoperatively.

**Conclusion:**

Most of the time acute SN responds well to conservative treatment; however rapid deterioration of symptoms or persistent severe pain should give suspicion of underlying secondary pathology.

## Background

Schmorl’s node (SN) is herniation of intervertebral disc into vertebral body through end plate defect. They first described by Christian Schmorl in 1927. SN are mostly asymptomatic and incidental findings on MRI; however sometimes they present like acute onset low back pain or acute exacerbation of chronic back pain after minor trauma [1, 2]. At that time they are called as acute SN. A number of theories have been proposed to explain the pathogenesis of SN which consist of developmental disease [3], degenerative disease [4], traumatic [5] or as part of autoimmune disorder [6]; still exact etiology of SN is unknown.

We present atypical presentation of acute symptomatic SN with infective pathology. There are no reports in the literature regarding the occurrence, imaging follow-up, treatment and outcome of symptomatic acute infective SN till date.

## Case presentation

### Case history

Sixty-seven years female patient presented to our hospital with complains of low back pain radiating to right buttock since 1 month with visual analogue scale (VAS) score 7. There was no associated fever or significant trauma in recent past. She had no other medical or surgical history in the past. Clinically, there was no tenderness over lumbar spine. Patient was neurologically intact.

### Investigations

**Fig. 1:**
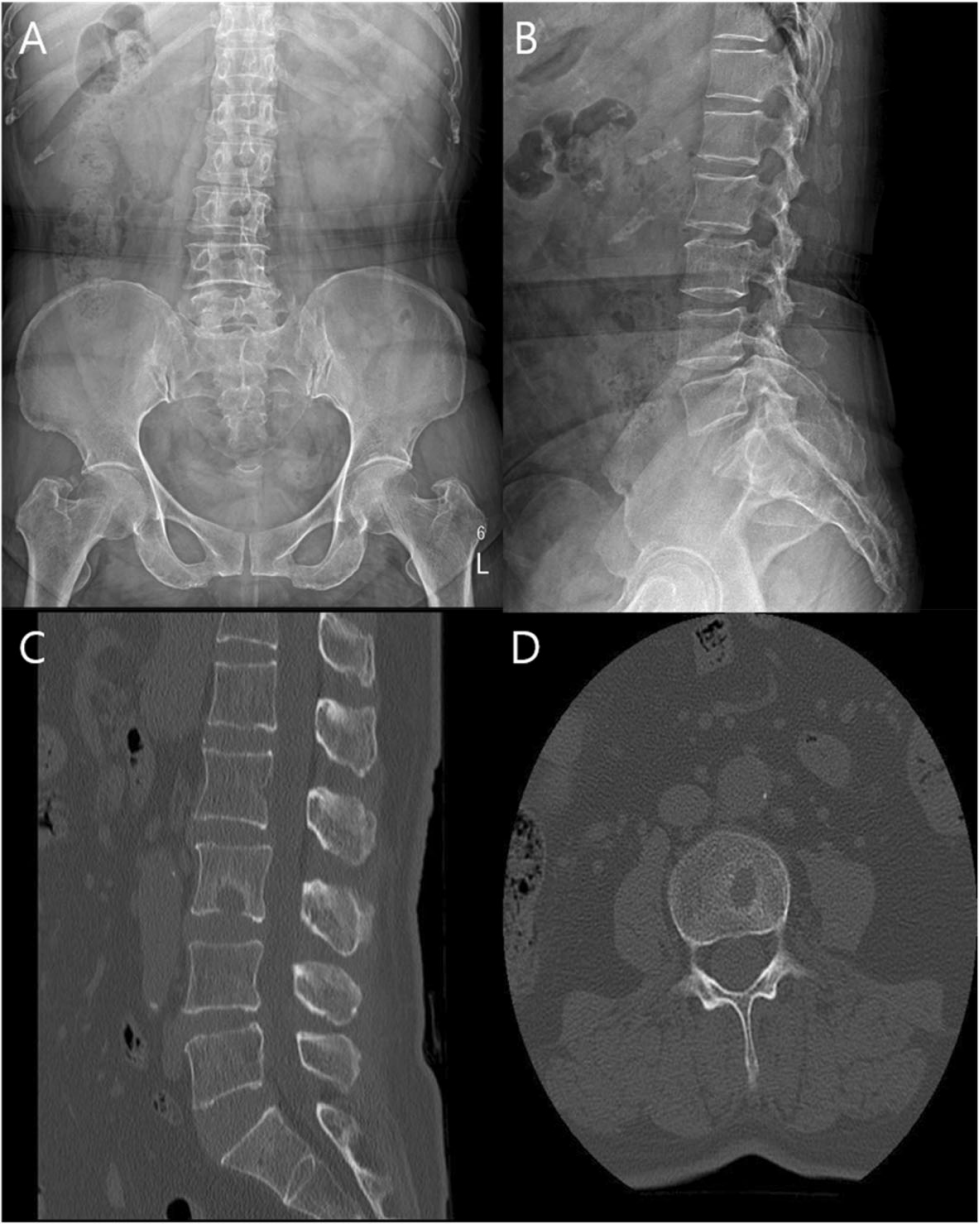
plain radiograph AP (**a**) and lateral view (**b**) showing mild degenerative scoliosis CT scan saggital (**c**) and axial (**d**) section showing schmorls node at inferior end plate of L3 vertebra with end plate defect

**Fig. 2:**
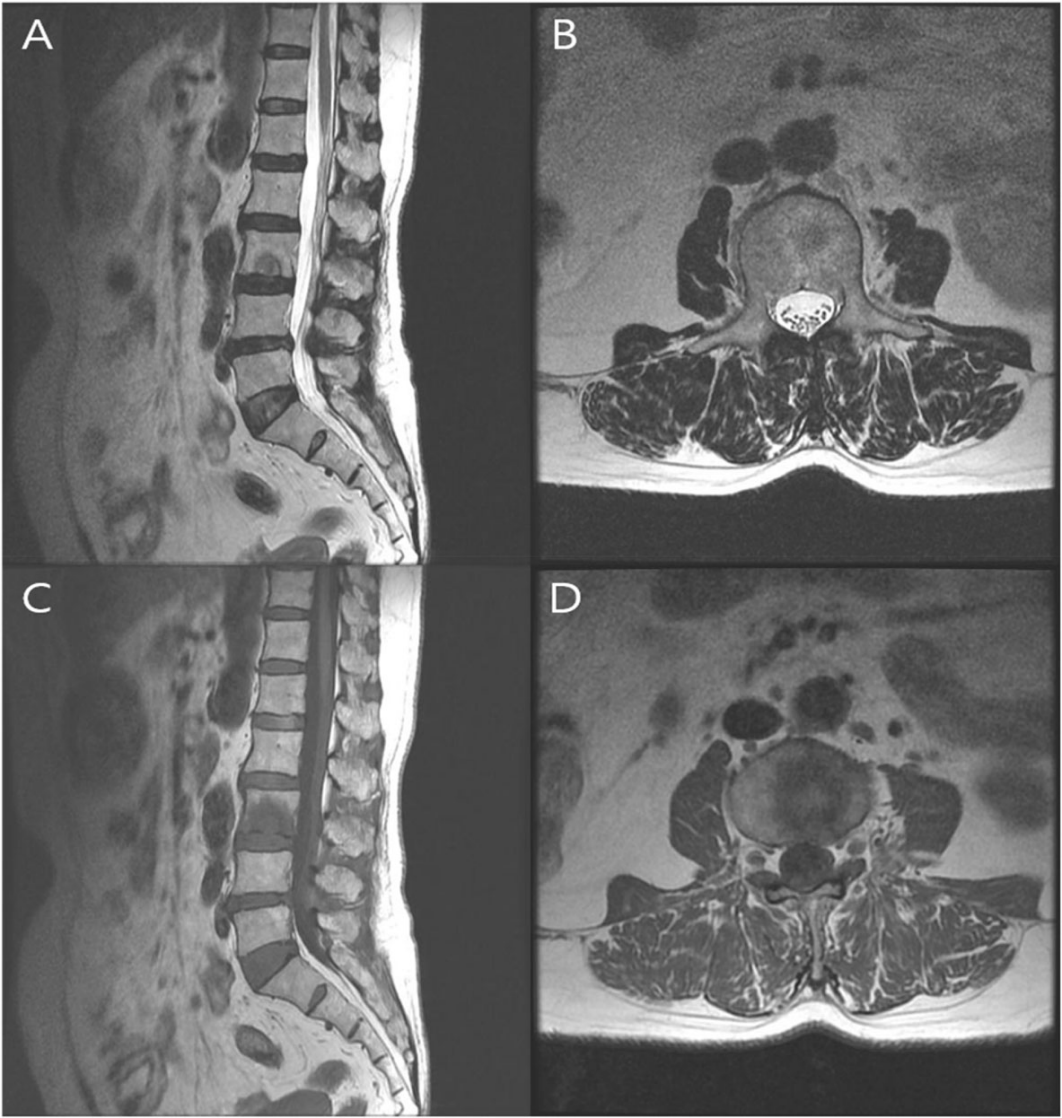
Initial MRI (**a**) T2 weighted sagittal image and (**b**) T2 weighted axial view showings Schmorl’s node with iso to high signal intensity, (**c**) T1 weighted sagittal image and (**d**) T1 weighted axial image showing low signal intensity

**Fig. 3:**
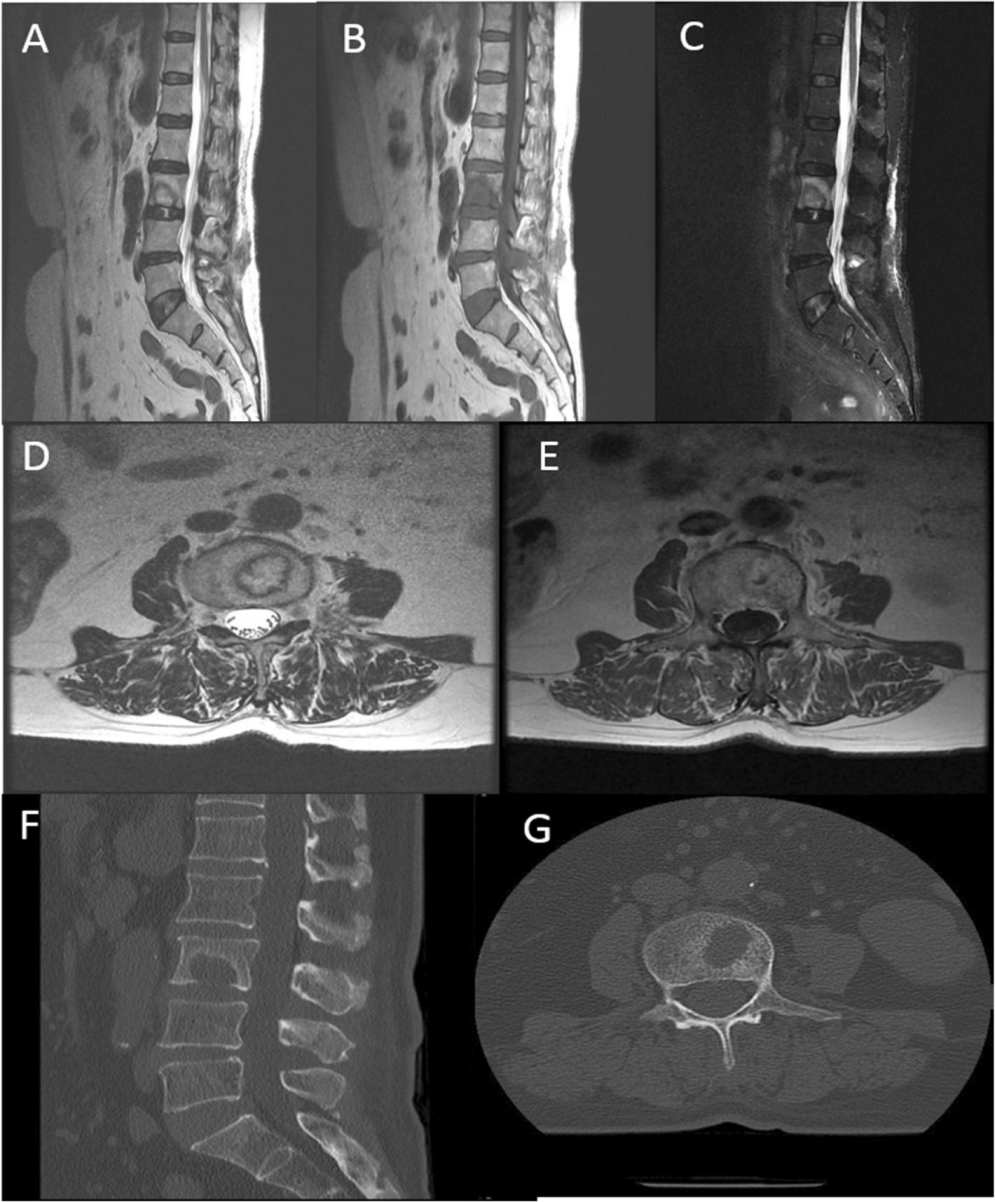
Repeat MRI (**a**, **b**) T1 weighted sagittal image and (**c**) STIR image showing increased signal intensity in L3 vertebra with anterio-superior part of L4. **b** Follow-up T2 weighted images showing decreased signal intensity suggestive of extensive marrow oedema. Repeat CT scan saggital (**f**) and axial (**g**) section showing increase in size of osteolytic lesion

A plain radiograph of lumbar spine was showing L4–5 narrowing of disc space with mild degenerative scoliosis. Dynamic plain radiograph does not show any features of instability. Computed tomography (CT) scan showed osteolytic bone lesion at inferior end plate of L3 vertebral body (Fig. 1). Initial magnetic resonance imagining (MRI) revealed an acute SN at lower end plate of L3 vertebral body without surrounding marrow signal changes (Fig. 2). A diagnosis of acute Schmorl’s node L3 vertebral body was made. Initial MRI and imaging findings of infected SN were difficult to suspect infective pathology; hence managed conservatively. Repeat subsequent magnetic resonance imaging (MRI) showed significant increase in size of lesion with focal signal changes in L3–4 disc and marrow changes in anterio-superior part of L4 vertebral body. A computed tomography (CT) at the same time also revealed increase in size of osteolytic lesion in L3 body (Fig. 3). Blood investigations revealed increase in the inflammatory markers suggestive of underlying infective or inflammatory pathology.

### Treatment

**Fig. 4:**
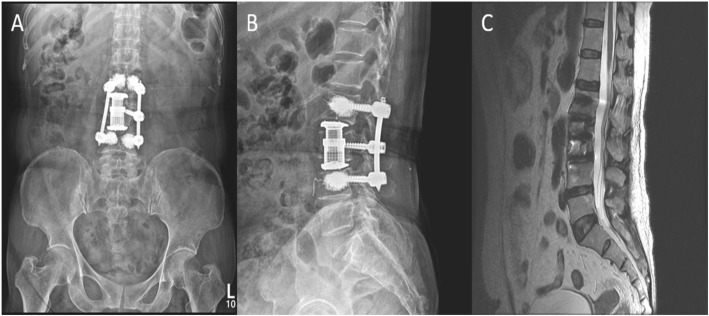
Post-operative 6 months radiograph: **a** AP view, **b** Lateral view, **c** 6 months follow-up MRI showing complete excision of lesion and complete resolution of osteolytic signal

We performed partial corpectomy L3 and expandable cage insertion through lateral retroperitoneal approach with posterior bone cement augmented percutaneous pedicle screw fixation L2-L4. Sample sent for histopathological examination and microbiology culture. Post operative MRI showed complete excision of lesion with persistent marrow signal changes at anterio-superior part of L4 vertebral body (Fig. 4). Histopathological report of tissue revealed acute granulomatous inflammation supporting diagnosis of pyogenic vertebral osteomyelitis; however, no organism was grown on microbiological culture. Sample also examined for the Acid fast bacillus, found negative. Empirical intravenous antibiotics (3rd generation cephalosporins + aminoglycosides) started from 1st post operative day and continued for 6 weeks. Patient was comfortable in post operative period with visual analogue scale score 3 at the time of discharge and improved during follow up visits. Inflammatory markers value also significantly reduced.

## Discussion and conclusion

Most of the SNs are asymptomatic and incidental finding [7]. It is reported to occur in 38 to 75% of population [4, 8]. it is predominantly found among males of European and American population [9]. The most common anatomical site of SNs to occur is thoracolumbar junction(T7-L1). It most commonly occurs in the superior end plate of lumbar vertebra and inferior end plate of thoracic vertebra [9–11].

Various theories have been proposed to explain the pathogenesis of SN in which widely accepted theory is axial load model proposed by Dar et al. [9] They proposed combination of increased range of movement, anteriorly located instantaneous axis of rotation and low disc thickness relative to vertebral body height in thoracic spine makes this region more vulnerable for stress and microfractures which accumulates over period of time to develop into SNs. Zhang et al. [6] postulated the immune system theory for the development of symptomatic SN proposed role of immune reaction to “non self” nucleus pulposus tissue which leads to influx of cytokines, inflammation in vertebral body and pain supported by an MRI study by Takahashi et al. [12] In our case, it is unclear if it developed secondary to weakening of the end plate from infection versus pre-existing SN acting as nidus for infectious bacteria to grow. Only after rapid progression of symptoms; more classical radiological features of spondylitis appeared.

Conventional radiograph has very limited value for diagnosing SNs. It is useful only in late stage of SN where sclerosis appear around lesion. These findings can be seen early and in more details on CT scan. Alternatively, Presence of bone oedema on Dual energy CT can suspect the infective/inflammatory pathology [13]. MRI is gold standard investigation for diagnosing SNs. it also differentiates between symptomatic and asymptomatic SN. symptomatic SNs show low signal intensity on T1 weighted image and high signal intensity on T2 weighted and short tau inversion recovery (STIR) images [12, 14, 15] as seen in our report; however, it is difficult to differentiate between benign degenerative bone disease and malignant infiltration or infection in early stages. Gadolinium contrast enhanced MRI can additionally differentiate between the bone oedema or soft tissue swelling from epidural abscess; however, it is contra indicated in patients with allergy to contrast and poor renal function. In the patients with poor renal fuction, diffusion weighted imaging (DWI) may be useful tool to differentiate between infective pathology from non infective cystic lesions. In late stages, MRI shows extensive marrow edema extending more than 2 adjacent vertebral body or structural collapse of vertebral body with paraspinal soft tissue shadows. Tubercular spondylodiscitis can be differentiated from bacterial spondylodiscitis by its rapid progression and sparing of disc space in early stage [16]. In our case, the SN seemed to be the site of infection because it was a singular lesion with the oedema centred around it, with worsening of oedema on subsequent imaging.

Conservative line of management always will be first line of management in symptomatic SN. Symptoms usually resolves within 2 to 6 months; however marrow edema on MRI gradually subsides over period of 3 to 12 months [17]. We also initially managed patients with analgesics and bed rest for 4 weeks; despite of which patients pain persisted. There are few case reports of symptomatic SN not responding to medical treatment are managed with surgical treatment. Various less invasive surgical modalities tried for treatment for symptomatic SNs which includes percutaneous vertebroplasty [18], tumor necrosis factor-alpha (TNF-alpha) [19], Rami communicans nerve block [20] successfully.

Peng et al. [21] did segmental fusion surgery (ALIF and PLIF) for severe low back pain not responding to conservative treatment due to SNs with overall fusion rate of 91%. Hasegawa et al. [22] performed retroperitoneal excision of an L3 vertebral body for SN. According to him patient improved significantly in post operative period. In our patient we performed excision of lesion with partial corpectomy and expandable cage insertion through lateral retroperitoneal approach.

**Fig. 5:**
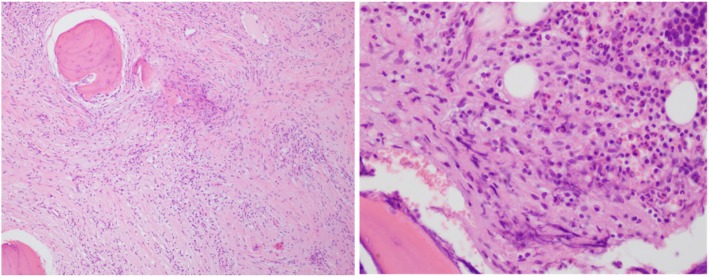
Histopathology slide (**a**) 100 HPF and (**b**) 400 HPF showing inflammatory cells infiltration with acute granulation tissue suggestive of pyogenic vertebral osteomyelitis

Typical Histopathological examination of pyogenic vertebral osteomyelitis shows inflammatory cell infiltration, vascular proliferations with granulation tissue, fibrosis, thrombosed blood vessels and bony necrosis depending upon stage of disease; as seen in current case report (Fig. 5). It was not always possible to isolate bacteria from microbiological culture. Depending on clinic radiological findings diagnosis of acute infective SN with vertebral osteomyelitis is made. Patient started on IV antibiotics for 6 weeks. Patient responded well to treatment with significant pain relief. Many case reports have been found in literature regarding degenerative symptomatic SNs; However, we could not find any reference related to acute infected SN in the literature. One case reported of tuberculous spondylitis which created diagnostic dilemma with acute symptomatic SN which eventually treated with anti-tubercular drugs [23].

In this study, we present the first report of infectious SN, which is very similar to acute non-infected Symptomatic SN at the beginning. Therefore, if infected SN is suspected based on laboratory studies Careful follow-up observation is essential. However, the clinical course along with MRI and CT findings and histological images are more suggestive of infective pathology instead of degeneration hence managed aggressively with excision of infective focus.

SN is always considered as one of the differential diagnosis of acute low back pain. Most of the time patient responds well to conservative treatment with analgesics and bed rest. Rapid deterioration of symptoms or persistent severe pain despite of conservative treatment should give suspicion of underlying another secondary pathology. It should be aggressively investigated and treated without further delay. MRI is investigation of choice for early diagnosis as well as to differentiate between symptomatic and asymptomatic SN. Currently there is no established treatment modality. Future investigations should address interventions for treating symptomatic SNs.

## Data Availability

The datasets used and/or analysed during the current study are available from the corresponding author on reasonable request.

## Abbreviations

SN: Schmorl’s node
MRI: Magnetic resonance imaging
CT: Computed tomography
ALIF: Anterior lumbar interbody fusion
PLIF: Posterior lumbar interbody fusion
